# Human Leukocyte Antigen DQB1 (HLA-DQB1) Polymorphisms and the Risk for Guillain-Barré Syndrome: A Systematic Review and Meta-Analysis

**DOI:** 10.1371/journal.pone.0131374

**Published:** 2015-07-23

**Authors:** Peng-Peng Jin, Li-Li Sun, Bo-Jun Ding, Na Qin, Bin Zhou, Feng Xia, Li Li, Li-Juan Liu, Xue-Dong Liu, Gang Zhao, Wen Wang, Yan-Chun Deng, Shuang-Xing Hou

**Affiliations:** 1 Centre for Medical Research and Innovation, Shanghai Pudong Hospital, Fudan University Pudong Medical Center, 2800 Gongwei Road, Shanghai 201399, China; 2 Department of Neurology, Xijing Hospital, Fourth Military Medical University, Xi’an 710032, China; 3 Unit of Evidence-Based Medicine, K. K. Leung Brain Research Centre, Fourth Military Medical University, Xi’an 710032, China; 4 Department of Neurology, Shanghai Pudong Hospital, Fudan University Pudong Medical Center, 2800 Gongwei Road, Shanghai 201399, China; Friedrich-Alexander University Erlangen, GERMANY

## Abstract

Guillain-Barré syndrome (GBS) is an autoimmune disorder of the peripheral nervous system. There is no consensus regarding reported associations between human leukocyte antigen DQB1 (HLA-DQB1) polymorphisms and the risk for developing GBS. Here, we evaluated possible associations between HLA-DQB1 polymorphisms and the risk for GBS using a meta-analysis. We searched PubMed for case-control genetic association studies for HLA-DQB1 polymorphisms (*020x, *030x, *040x, *050x, and *060x) and the risk for GBS. Fixed-effect meta-analytical methods were used for the outcome measure and subgroup analyses. Estimated odds ratios (ORs) and 95% confidence intervals (CIs) were used to investigate the associations between HLA-DQB1 polymorphisms and the risk for GBS. Nine case-control studies involving 780 cases of GBS and 1353 controls were identified in the current study. The meta-analysis demonstrated no significant associations between HLA-DQB1 polymorphisms and the risk for GBS in Asian and Caucasian populations. There were two associations that approached significance: HLA-DQB1*030x in Asian patients (*P* = 0.07; OR: 0.76, 95% CI: 0.57–1.03) and HLA-DQB1*060x in all patients (*P* = 0.08; OR: 1.48, 95% CI: 0.96–2.29). Additional studies with larger sample sizes are required to establish a definitive assessment of the contribution of HLA-DQB1 polymorphisms to GBS risk.

## Introduction

Guillain-Barré syndrome (GBS) is an autoimmune disorder of the peripheral nervous system. GBS is initiated by an abnormal response to an infectious pathogen and is characterized by progressive flaccid paralysis and loss of reflexes. GBS is a heterogeneous disorder with several subtypes, including acute inflammatory demyelinating polyneuropathy (AIDP), acute motor axonal neuropathy (AMAN), and acute motor sensory axonal neuropathy (AMSAN) [1]. The prevalence of the subtypes varies regionally; AIDP is the most common subtype in the West, while AMAN and AMSAN are more common in Asia [2]. Infections including Cytomegalovirus, Epstein-Barr virus, and *Campylobacter jejuni* have all been linked to GBS [1,2]. *C*. *jejuni* infection is the most common infectious trigger and is present in 25–40% of GBS cases [2]. However, the annual incidence of GBS is very small, 0.6–4 cases per 100,000 population, which equates to approximately 1 out of every 1,000–5,000 cases of *C*. *jejuni* infection [2,3]. The rarity of GBS cases, even in patients with *C*. *jejuni*, suggests that other factors determine whether a patient will develop GBS following infection.

A candidate risk factor for host susceptibility to GBS is the human leukocyte antigen (HLA) haplotype. HLAs are highly polymorphic gene clusters that affect immune responses to infection [4–6] and are implicated in autoimmune diseases [7,8]. While associations with major histocompatibility complex (MHC) class I antigens have been documented, these are often secondary to associations with MHC class II antigens [9]. HLA class II molecules play an important role in activating immune responses and help recognize self or foreign antigens [10]. The HLA class II genes, especially the highly polymorphic HLA-DQ alleles, may mediate the autoimmune responses that contribute to GBS [Klein 2000].

Several reports have described the relationship between class II HLA-DQ polymorphisms and the risk for various autoimmune diseases, including GBS [11]. Associations between the HLA-DQB1*03 and HLA-DQB1*060x polymorphisms and the risk for GBS have been investigated previously in patients of different ethnicities. In patients from northern China, HLA alleles are differentially distributed in two forms of GBS (AIDP and AMAN) [12]. In Caucasian GBS patients, an association between the HLA-DQB1*03 polymorphism and *C*. *jejuni* infection has been reported [13]; and in Indian patients, the HLA-DQB1*060x polymorphism has been associated with risk for GBS [14]. However, to date, there is no consensus regarding whether GBS is linked to HLA type. Previous work has been limited by small sample sizes, imprecise HLA typing by current standards, and ethnic and geographical differences across studies. The objective of this meta-analysis was to evaluate the relationship between HLA-DQB1 polymorphisms and the risk for GBS based on currently available case-control studies.

## Materials and Methods

This meta-analysis was performed following the Quality of Reporting of Meta-analyses (QUORUM) guidelines [15] and the recommendations of the Cochrane Collaboration [16].

### Search strategy

We conducted a systematic search of PubMed (1950–December, 2014) using the following key words: “Human leukocyte antigen*” OR “HLA*” AND “allele” OR “polymorphism” OR “genotype” AND “Guillain-Barré syndrome” OR “acute infiammatory demyelinating polyneuropathy” OR “acute motor axonal neuropathy” OR “acute motor sensory axonal neuropathy.” Additional information was retrieved by a hand search of the reference lists of relevant articles. We repeated the search using the terms “DQB1*01”, “DQB1*02”, “DQB1*03”, “DQB1*04”, “DQB1*05”, and “DQB1*06” in place of the more general terms HLA or human leukocyte antigen. No additional unique records were identified, so we proceeded using only the search results from our original query.

### Inclusion and exclusion criteria

We included studies that (1) examined the potential association between HLA polymorphisms and the risk for GBS; (2) had a case-control design; (3) selected controls from healthy people or the community; and (4) contained sufficient information on genotype frequency. To achieve statistical power, we only considered polymorphisms that were reported in more than two publications. Our search was limited to articles written in English.

We excluded publications that (1) were reviews, editorials, or comments; (2) did not evaluate the association between HLA polymorphisms and the risk for GBS; (3) did not report genotype frequency or the relevant information could not be obtained by contacting the study authors; (4) reported on polymorphisms that were examined in less than two publications; (5) were duplicate studies; or (6) were basic science or animal studies.

### Study selection

Three review authors (SX Hou, LL Sun, and PP Jin) independently examined the titles and abstracts to select eligible studies. We retrieved the full-text articles of potentially relevant studies. Where data sets were overlapping or duplicated, only the most recent information and the largest study size were included. Three review authors (SX Hou, LL Sun, and PP Jin) independently examined the full-text records to determine which studies met the inclusion criteria. Disagreements regarding study selection were resolved through group discussion and consensus.

### Data extraction

Four review authors (SX Hou, LL Sun, JB Dong, and LJ Liu) independently extracted information from eligible studies. Data included the following: first author’s name, year of publication, patient population, number of cases and controls, genotyping protocols, and diagnostic criteria. The final analysis included event counts from cases and controls for allele typing out to two digits, where necessary, derived from more extensive typing. Disagreements regarding data extraction were resolved through group discussion and consensus.

### Subgroup analyses

Subgroup analyses were performed by stratifying patients according to ethnicity (Asian and Caucasian). One full-text study [17] was excluded from the subgroup analyses as the participants were neither Asian nor Caucasian.

### Statistical methods

Statistical analyses were conducted using Review Manager 5.1 software (Cochrane Collaboration, http://ims.cochrane.org/revman/download). Odds ratios (ORs) and their 95% confidence intervals (95% CIs) were used to estimate the strengths of the associations between HLA polymorphisms and the risk for GBS. The Z test determined the significance of the pooled OR, and a P value less than 0.05 was considered significant. A random-effects model was used to pool studies with significant heterogeneity (Der Simonian and Laird method [18]) as determined by Cochran’s χ^2^-based Q statistic test (*P* ≤ 0.10) [19] and the inconsistency index (*I* ^*2*^ ≥ 50%) [20]. Otherwise, a fixed-effects model (Mantel-Haenszel method [21]) was used to calculate the pooled ORs and P values.

Publication bias was assessed using a funnel plot of effect estimates against their standard errors (SEs), Begg-Mazumdar rank correlation, and Egger’s test [22,23].

## Results

**Fig 1:**
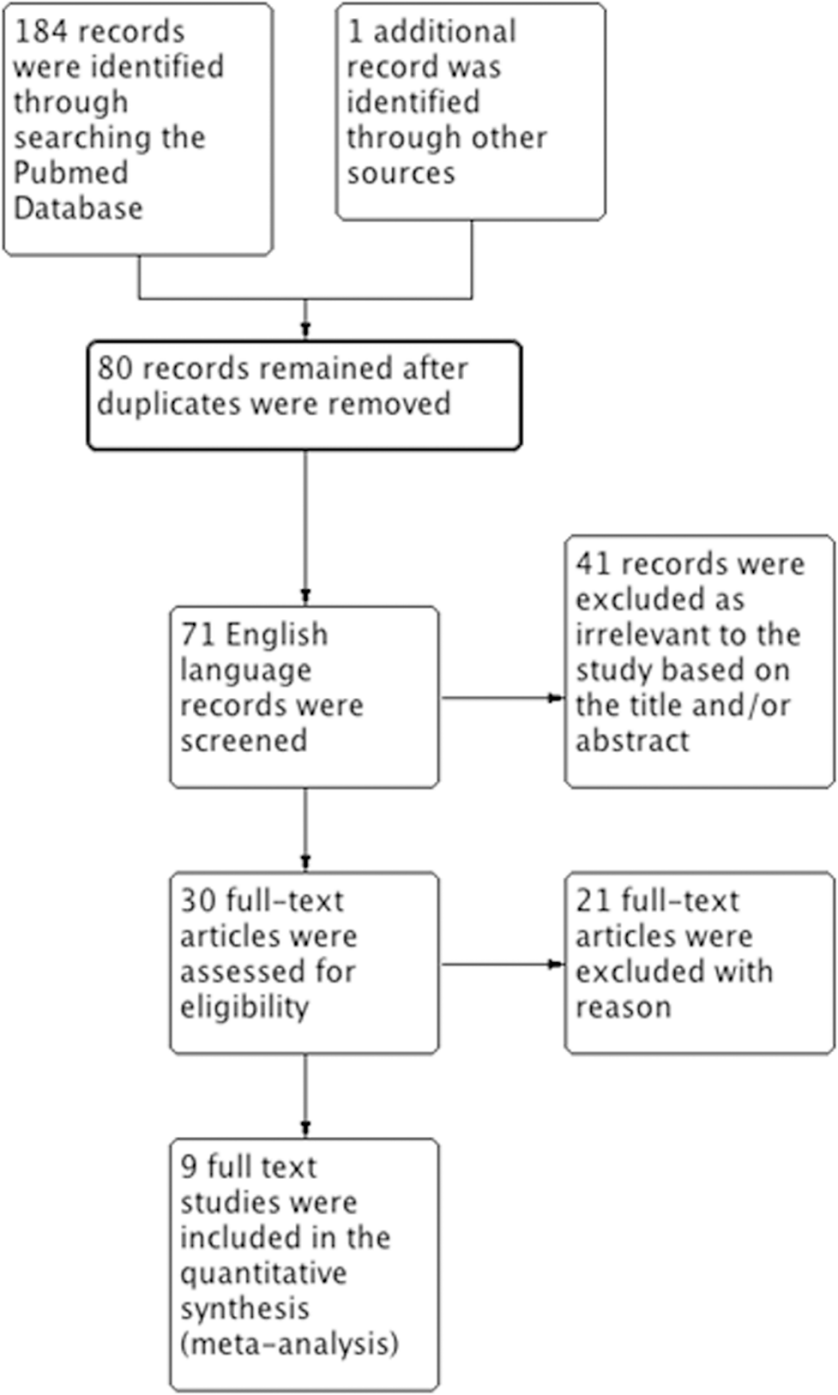
Flow chart of article screening and selection process.

The searches identified 184 articles, of which 80 were unique records and 71 were in English. Based on screening the titles and abstracts, 30 studies were identified as potentially eligible for inclusion. After analyzing the full-text articles, 21 studies were excluded and nine [12–14, 17, 24–28] were found eligible for inclusion according to our criteria for this review (Fig 1).

### Included studies

The characteristics of the included studies are shown in Table 1. Of these studies, five [12, 14, 25, 26, 28] were conducted in Asian populations, three were conducted in Caucasian populations [13, 24, 27], and one was conducted in an Arabic population [17]. All of the studies were population-based, and the controls were selected from healthy subjects.

**Table 1:** Included Study Characteristics.

Study (Author, year)	Methods	Participants	Outcomes	Ethnicity	Country	Diagnostic Criteria
Rees 1997^a^	DNA was extracted from peripheral blood and the DQB1 alleles were identified by PCR.	GBS cases = 97; Healthy Controls = 100	DQB1*020x DQB1*030x DQB1*040x DQB1*050x DQB1*0601	Caucasian	England/Wales	Asbury and Cornblath
Koga 1998	DQB1 alleles were typed using the PCR-dependent preferential heteroduplex formation assay.	GBS cases = 35; Healthy Controls = 112	DQB1*020x DQB1*030x DQB1*040x DQB1*050x DQB1*060x	Asian	Japan	Asbury and Cornblath
Ma 1998	HLA-DQB1 allele typing was performed using a modified PCR-restriction fragment length polymorphism method combined with group-specific primers.	GBS cases = 81; Healthy Controls = 87	DQB1*030x DQB1; 040x DQB1*050x DQB1*060x	Asian	Japan	Asbury and Cornblath
Li 2000	DNA was extracted from white blood cells and the HLA typing was performed using PCR with sequence-specific primers.	GBS cases = 47; Healthy Controls = 50	DQB1*020x DQB1*030x DQB1*040x DQB1*050x DQB1*060x	Asian	China	Asbury and Cornblath
Magira 2003^b^	Genomic DNA was obtained and HLA typing performed by PCR with sequence-specific primers.	GBS cases = 72 (47 AMAN, 25 AIDP); Healthy Controls = 97	DQB1*020x DQB1*030x DQB1*040x DQB1*050x DQB1*060x	Asian	China	Asbury and Cornblath
Geleijns 2005	Genomic DNA was extracted from whole blood samples and the HLA type was determined at the two-digit level using PCR with sequence-specific primers.	GBS cases = 164; Healthy Controls = 207	DQB1*02 DQB1*03 DQB1*04 DQB1*05 DQB1*06	Caucasian	Netherlands	Asbury and Cornblath
McCombe 2006	Genomic DNA was extracted from whole blood and the HLA-DQB alleles were typed using Dynal low-resolution SSP kits.	GBS cases = 74; Healthy Controls = 158	DQB1*050x DQB1*060x	Caucasian (n = 73) and Asian (n = 1)	Australia	Asbury and Cornblath
Sinha 2010^c^	Genomic DNA was isolated from whole blood and the HLA type at the HLA-II DQB1 locus was determined at the two-digit level using PCR with sequence-specific primers.	GBS cases = 54; Healthy Controls = 202	DQB1*0201 DQB1*030x DQB1*040x DQB1*050x DQB1*060x	Asian	India	Asbury and Cornblath; subtype diagnosis based on Hadden *et al*.
Fekih-Mrissa 2014	Genomic DNA was extracted from peripheral blood samples, and low-resolution HLA typing was performed using Micro SSP DNA typing trays DRB/DQB.	GBS cases = 38; Healthy Controls = 100	DQB1*020x DQB1*030x DQB1*050x DQB1*060x	Arabic	Tunisia	Asbury and Cornblath

^a^ Blood samples were obtained from 103 GBS cases for a previous study, but only 93 were available for the analyses in this study. An additional four cases were recruited between the end of the first study and the analysis for this study.

^b^ All participants were diagnosed with GBS; however, for this study they were divided into the AMAN and AIDP forms of GBS.

^c^ HLA typing was performed on a subset of randomly selected GBS cases from a larger cohort (n = 80) and compared to a pool of 202 healthy controls rather than the pool of 80 matched healthy controls recruited for the case/control section of the study.

Data pertaining to the frequency of HLA-DQB1 alleles to at least two digits in GBS patients and healthy controls were available for-DQB1*020x,-DQB1*030x,-DQB1*040x,-DQB1*050x, and-DQB1*060x. Six studies with 448 cases and 818 controls included relevant data for the–DQB1*020x polymorphism [12–14, 17, 24, 28]. Eight studies with 680 cases and 1009 controls included data for the–DQB1*030x polymorphism [12–14, 17, 24–26, 28]. Seven studies with 598 cases and 953 controls contained data for the–DQB1*040x polymorphism [12–14, 24–26, 28]. Nine studies with 780 cases and 1353 controls included data for the–DQB1*050x and–DQB1*060x polymorphisms [12–14, 17, 24–28].

### Excluded studies

Of the 30 studies that were relevant to HLA polymorphisms and the risk for GBS, 21 were excluded (Table 2). Of these studies, 13 were excluded because the HLA typing was insufficiently precise, six were excluded because of insufficient data due to a low patient number (five were family pairs), and one was excluded because it was an animal study.

**Table 2:** Excluded Studies.

Study (Author, year)	Citation	Reason(s) for Exclusion
Adams 1977	Lancet. 1977 Sep 3;2(8036):504–5.	HLA typing was insufficient^a^
Stewart 1978	Ann Neurol. 1978 Sep;4(3):285–9.	HLA typing was insufficient
Latovitzki 1979	Neurology. 1979 May;29(5):743–5.	HLA typing was insufficient and study design was not case-control
Gorodezky 1983	J Neuroimmunol. 1983 Feb;4(1):1–7.	HLA typing was insufficient and included only HLA-DR alleles
Kaslow 1984	Neurology. 1984 Feb;34(2):240–2.	HLA typing was insufficient and included only HLA-A,-B, and-C alleles
Hafez 1985	J Neurogenet. 1985 Sep;2(4):285–90.	HLA typing was insufficient
Winer 1988	J Neuroimmunol. 1988 Apr;18(1):13–6.	HLA typing was insufficient
Hillert 1991	J Neuroimmunol. 1991 Jan;31(1):67–72.	HLA typing was insufficient
Yuki 1992	Muscle Nerve. 1992 Aug;15(8):968–9.	HLA typing was insufficient and study design was not case-control
Davidson 1992	J Neurol Neurosurg Psychiatry. 1992 Jun;55(6):508–9.	Familial study with insufficient genetic data
Piradov 1995	Neurology. 1995 Jul;45(7):1419–20.	HLA typing was insufficient and included only HLA-DR1 and-DR6 alleles
Chiba 1995	J Neuroimmunol. 1995 Aug;61(1):85–8.	HLA typing was insufficient
Monos 1997	J Infect Dis. 1997 Dec;176 Suppl 2:S180-2.	Only HLA-DR alleles were characterized
Grigg 1998	J Clin Neurosci. 1998 Apr;5(2):169–71.	Insufficient genetic data^b^
Wilmshurst 1999	Eur J Neurol. 1999 Jul;6(4):499–503.	Familial study with insufficient genetic data
Ang 2000	J Neuroimmunol. 2000 Nov 1;111(1–2):229–33.	Familial study with insufficient genetic data
de Graaf 2004	J Neuroimmunol. 2004 Oct;155(1–2):73–84.	Animal Study
Senanayake 2010	Ceylon Med J. 2010 Dec;55(4):135–6.	Familial study with insufficient genetic data
Barzegar 2012	Ann Indian Acad Neurol. 2012 Oct;15(4):299–302. doi: 10.4103/0972-2327.104341.	Familial study with insufficient genetic data
Blum 2013	J Neuroimmunol. 2014 Feb 15;267(1–2):92–6. doi: 10.1016/j.jneuroim.2013.12.007. Epub 2013 Dec 12.	Only HLA-A,-B, and-C alleles were typed
Hasan 2014	Neurosciences (Riyadh). 2014 Oct;19(4):301–5.	Only HLA-DR alleles were characterized

^a^ Insufficient HLA typing was considered for any analysis that was not able to identify the HLA allele to at least two digits.

^b^ Studies with insufficient genetic data included only 2–3 GBS cases, were usually family groupings, and did not have a case-control design.

### HLA DQB1 polymorphisms and the risk for GBS

#### HLA-DQB1*020x polymorphism

**Fig 2:**
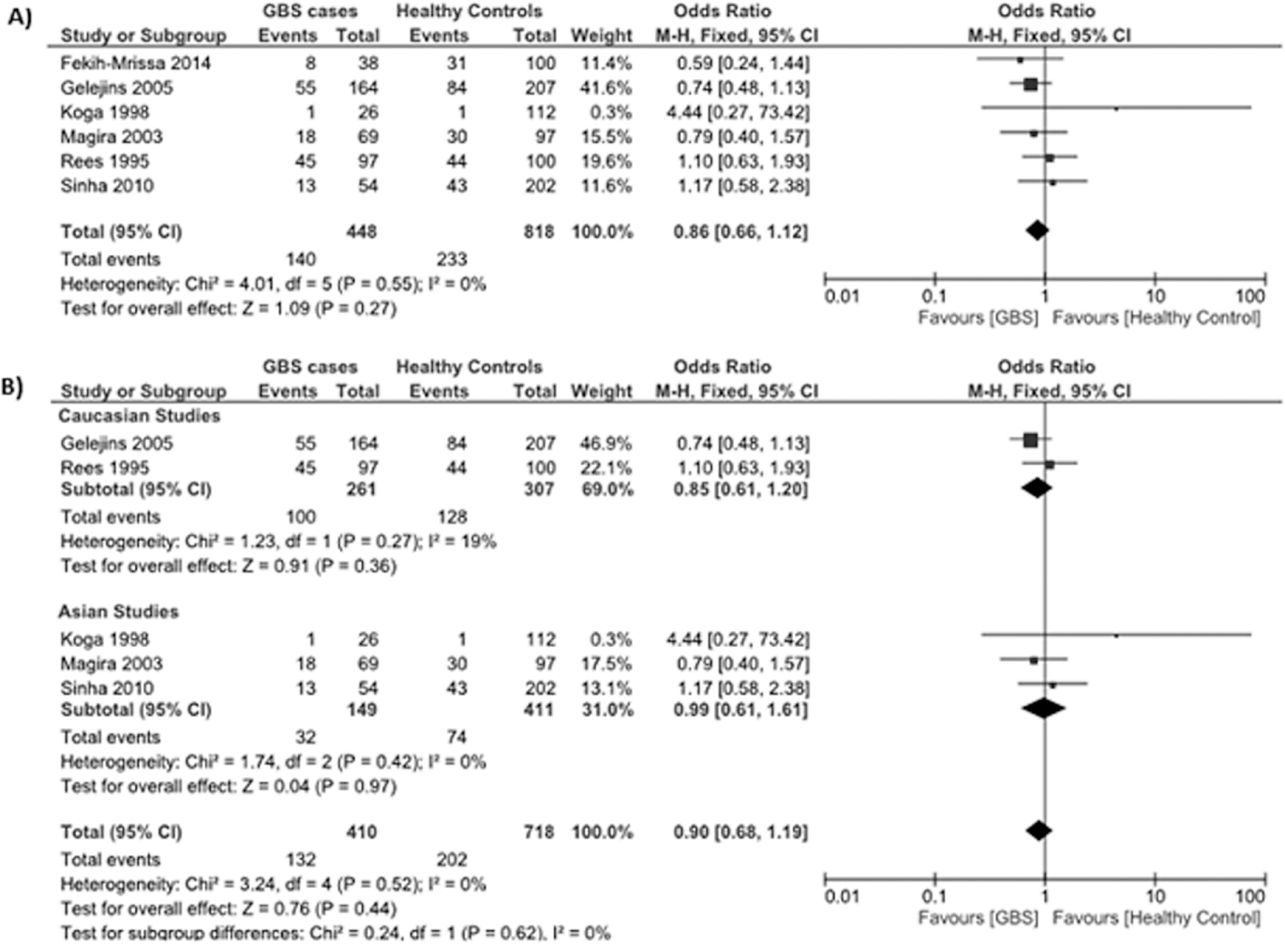
Association between the HLA-DQB1*020x polymorphism and the risk for GBS. (**A**) Six studies describe the association between the HLA-DQB1*020x polymorphism and the risk for GBS in a mixed Asian, Caucasian, and Arabic population. (**B**) Subgroup analysis examining the relationship between HLA-DQB1*020x and GBS risk in Caucasian (*top*) and Asian (*bottom*) populations.

Data reporting on the HLA-DQB1*020x polymorphism are described in six case-control studies [12–14, 17, 24, 28]. Three studies were conducted in Asian populations [12, 14, 28], two studies were conducted in Caucasian populations [13, 24], and one study was conducted in an Arabic population [17]. The meta-analysis demonstrated no significant association between the HLA-DQB1*020x polymorphism and the risk for GBS (OR: 0.86, 95% CI: 0.66–1.12; *P* = 0.27) (Fig 2A). There was no evidence of significant heterogeneity between studies (*P* = 0.55, *I* ^*2*^ = 0%).

Subgroup analyses stratified by ethnicity (Asian and Caucasian) demonstrated no significant association between the HLA-DQB1*020x polymorphism and the risk for GBS in Asians (OR: 0.99, 95% CI: 0.68–1.61; *P* = 0.97) or Caucasians (OR: 0.85, 95% CI: 0.61–1.61; *P* = 0.36) (Fig 2B).

#### HLA-DQB1*030x polymorphism

**Fig 3:**
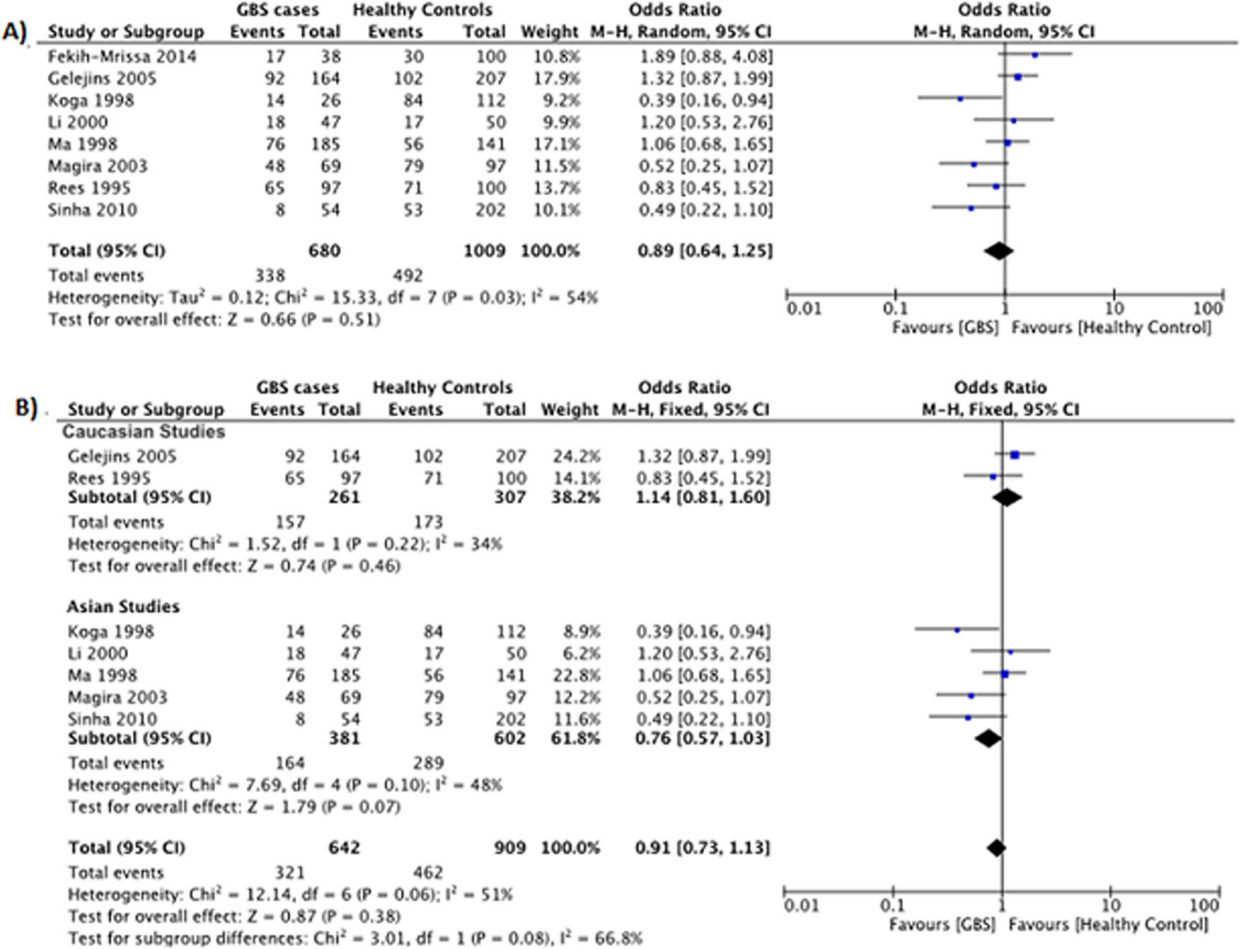
Association between the HLA-DQB1*030x polymorphism and the risk for GBS. (**A**) Eight studies describe the association between the HLA-DQB1*030x polymorphism and the risk for GBS in a mixed Asian, Caucasian, and Arabic population. (**B**) Subgroup analysis examining the relationship between HLA-DQB1*030x and GBS risk in Caucasian (*top*) and Asian (*bottom*) populations.

Data reporting on the HLA-DQB1*030x polymorphism are described in eight case-control studies [12–14, 17, 24–26, 28]. Five studies were conducted in Asian populations [12, 14, 25, 26, 28], two studies were conducted in Caucasian populations [13, 24], and one study was conducted in an Arabic population [17]. The meta-analysis demonstrated no significant association between the HLA-DQB1*030x polymorphism and the risk for GBS (OR: 0.89, 95% CI: 0.64–1.25; *P* = 0.51) (Fig 3A). However, there was evidence of significant heterogeneity between studies (*P* = 0.03, *I* ^*2*^ = 54%).

Subgroup analyses stratified by ethnicity (Asian or Caucasian) demonstrated no significant association between the HLA-DQB1*030x polymorphism and the risk for GBS in Asians (OR: 0.76, 95% CI: 0.73–1.13; *P* = 0.07) or Caucasians (OR: 1.14, 95% CI: 0.81–1.60; *P* = 0.46) (Fig 3B). There was no evidence of significant heterogeneity between studies within the Asian (*P* = 0.10, *I* ^*2*^ = 48%) or Caucasian (*P* = 0.22, *I* ^*2*^ = 34%) subgroups.

#### HLA-DQB1*040x polymorphism

**Fig 4:**
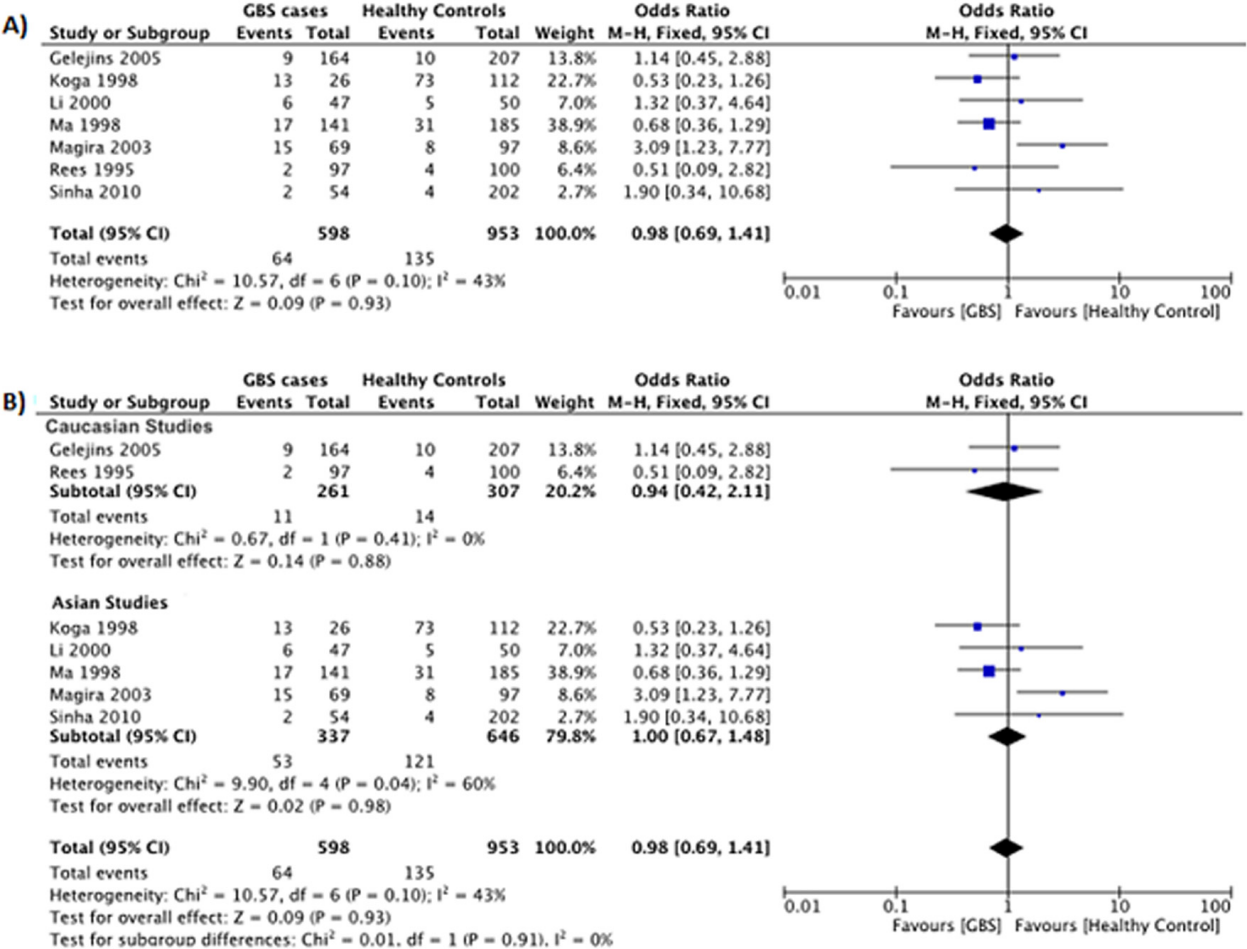
Association between the HLA-DQB1*040x polymorphism and the risk for GBS. (**A**) Seven studies describe the association between the HLA-DQB1*040x polymorphism and the risk for GBS in a mixed Asian and Caucasian population. (**B**) Subgroup analysis examining the relationship between HLA-DQB1*040x and GBS risk in Caucasian (*top*) and Asian (*bottom*) populations.

Data reporting on the HLA-DQB1*040x polymorphism are described in seven case-control studies [12–14, 24–26, 28]. Five studies were conducted in Asian populations [12, 14, 25, 26, 28], and two studies were conducted in Caucasian populations [13, 24]. The meta-analysis demonstrated no significant association between the HLA-DQB1*040x polymorphism and the risk for GBS (OR: 0.98, 95% CI: 0.69–1.41; *P* = 0.93) (Fig 4A). There was no evidence of significant heterogeneity between studies (*P* = 0.10, *I* ^*2*^ = 43%).

Subgroup analyses stratified by ethnicity demonstrated no significant association between the HLA-DQB1*040x polymorphism and the risk for GBS in Asians (OR: 0.98, 95% CI: 0.69–1.41; *P* = 0.93) or Caucasians (OR: 0.94, 95% CI: 0.42–2.11; *P* = 0.88) (Fig 4B). However, there was evidence of significant heterogeneity between studies in the Asian subgroup (*P* = 0.04, *I* ^*2*^ = 60%).

#### HLA-DQB1*050x polymorphism

**Fig 5:**
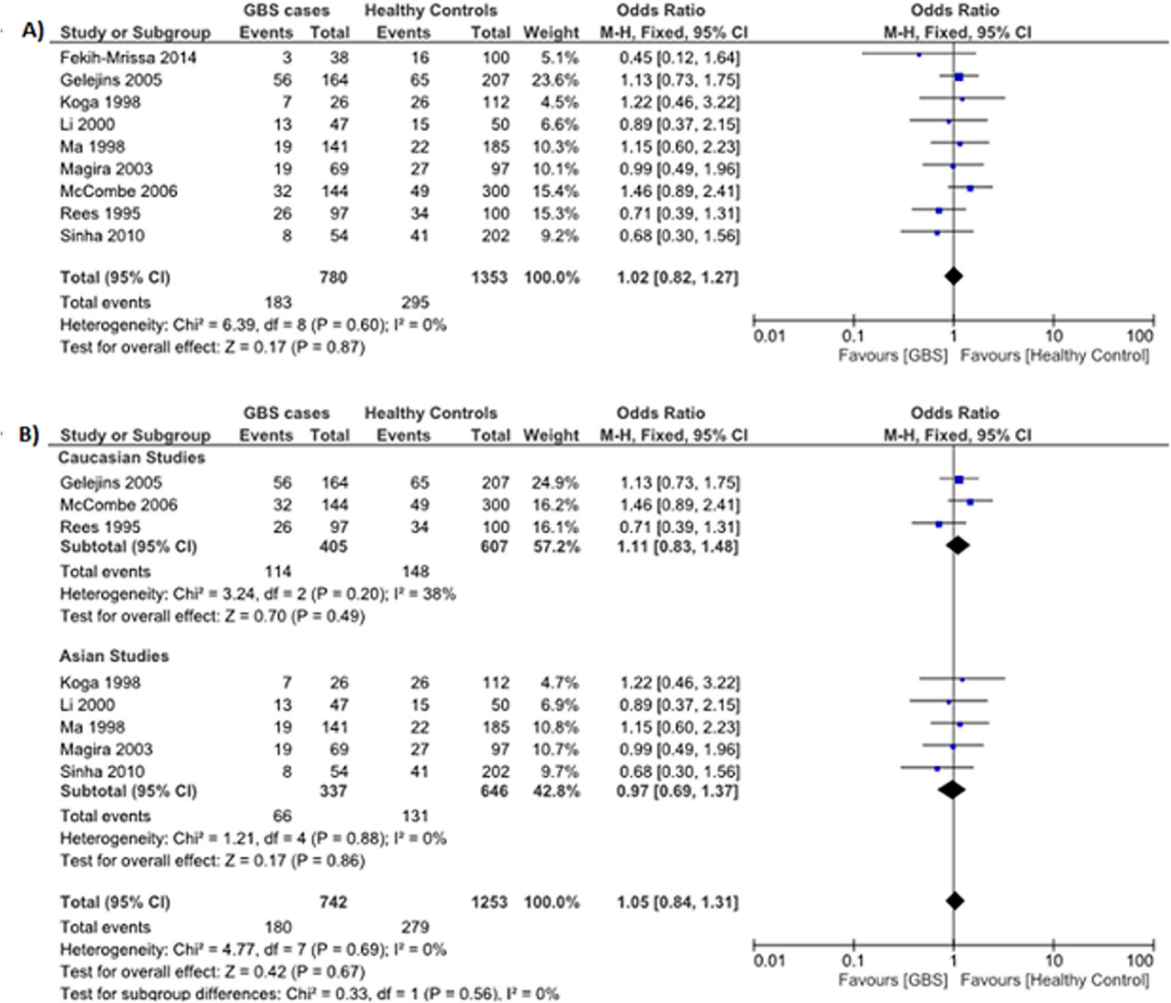
Association between the HLA-DQB1*050x polymorphism and the risk for GBS. (**A**) Nine studies describe the association between the HLA-DQB1*050x polymorphism and the risk for GBS in a mixed Asian, Caucasian, and Arabic population. (**B**) Subgroup analysis examining the relationship between HLA-DQB1*050x and GBS risk in Caucasian (*top*) and Asian (*bottom*) populations.

Data reporting on the HLA-DQB1*050x polymorphism are described in nine case-control studies [12–14, 17, 24–28]. Five studies were conducted in Asian populations [12, 14, 25, 26, 28], three studies were conducted in Caucasian populations [13, 24, 27], and one study was conducted in an Arabic population [17]. The meta-analysis demonstrated no significant association between the HLA-DQB1*050x polymorphism and the risk for GBS (OR: 1.02, 95% CI: 0.82–1.27; *P* = 0.87) (Fig 5A). There was no evidence of significant heterogeneity between studies (*P* = 0.60, *I* ^*2*^ = 0%).

Subgroup analyses stratified by ethnicity (Asian and Caucasian) demonstrated no significant association between the HLA-DQB1*050x polymorphism and the risk for GBS in Asians (OR: 0.97, 95% CI: 0.69–1.37; *P* = 0.86) or Caucasians (OR: 1.11, 95% CI: 0.83–1.48; *P* = 0.49) (Fig 5B).

#### HLA-DQB1*060x polymorphism

**Fig 6:**
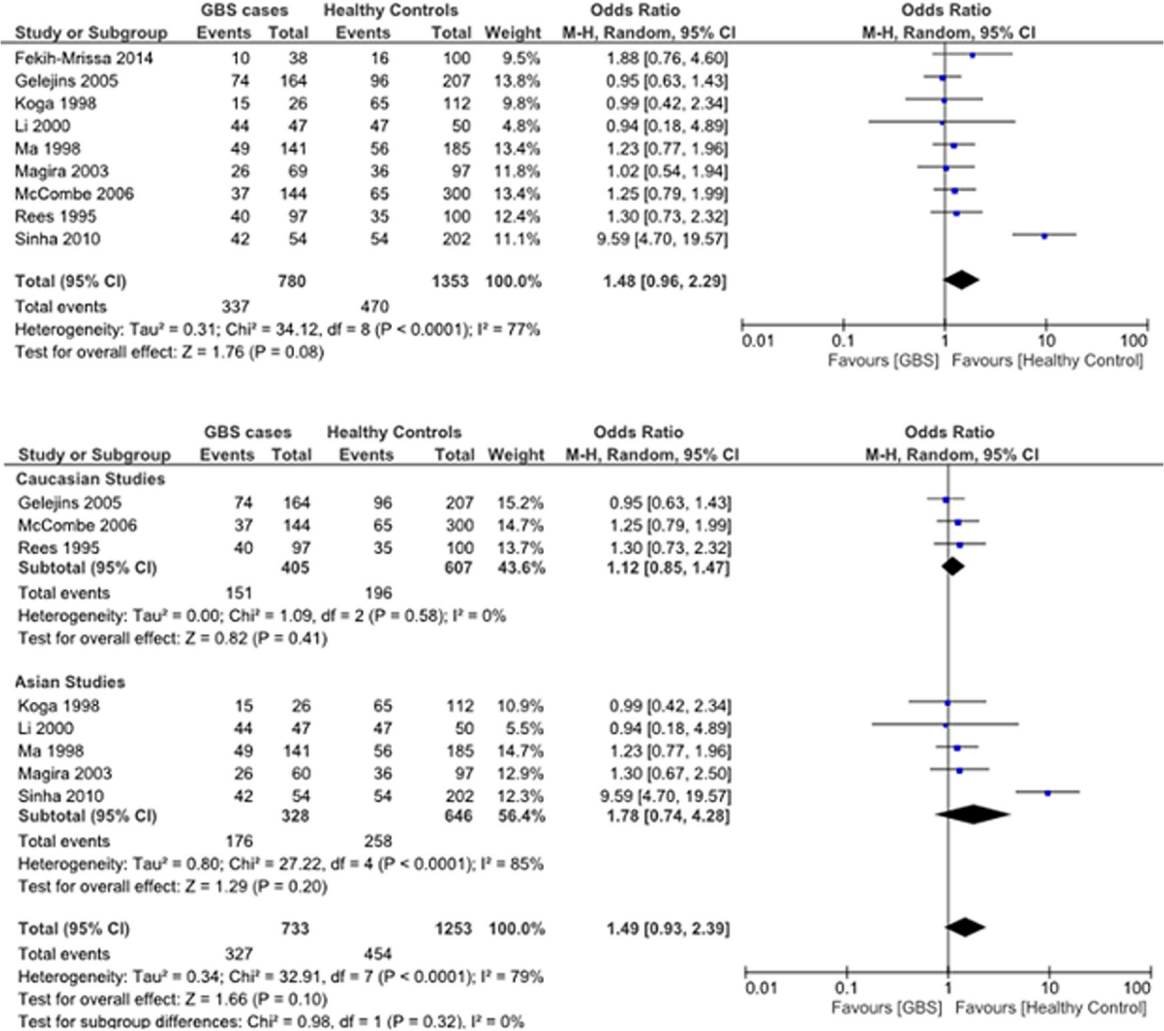
Association between the HLA-DQB1*060x polymorphism and the risk for GBS. (**A**) Nine studies describe the association between the HLA-DQB1*060x polymorphism and the risk for GBS in a mixed Asian, Caucasian, and Arabic population. (**B**) Subgroup analysis examining the relationship between HLA-DQB1*060x and GBS risk in Caucasian (*top*) and Asian (*bottom*) populations.

Data reporting on the HLA-DQB1*060x polymorphism are described in nine case-control studies [12–14, 17, 24–28]. Five studies were conducted in Asian populations [12, 14, 25, 26, 28], three studies were conducted in Caucasian populations [13, 24, 27], and one study was conducted in an Arabic population [17]. The meta-analysis demonstrated no significant association between the HLA-DQB1*060x polymorphism and the risk for GBS (OR: 1.48, 95% CI: 0.96–2.29; *P* = 0.08) (Fig 6A). There was proof of significant heterogeneity between studies (*P* < 0.0001, *I* ^*2*^ = 77%). The heterogeneity was due largely to the study by Sinha *et al*., which showed a significant increase in the frequency of the HLA-DQB1*060x allele in GBS patients compared to healthy controls [17].

Subgroup analyses stratified by ethnicity (Asian and Caucasian) demonstrated no significant association between the HLA-DQB1*060x polymorphism and the risk for GBS in Asians (OR: 1.78, 95% CI: 0.74–4.28, *P* = 0.20) or Caucasians (OR: 1.12, 95% CI: 0.85–1.47, *P* = 0.41) (Fig 6B).

#### Publication bias

**Fig 7:**
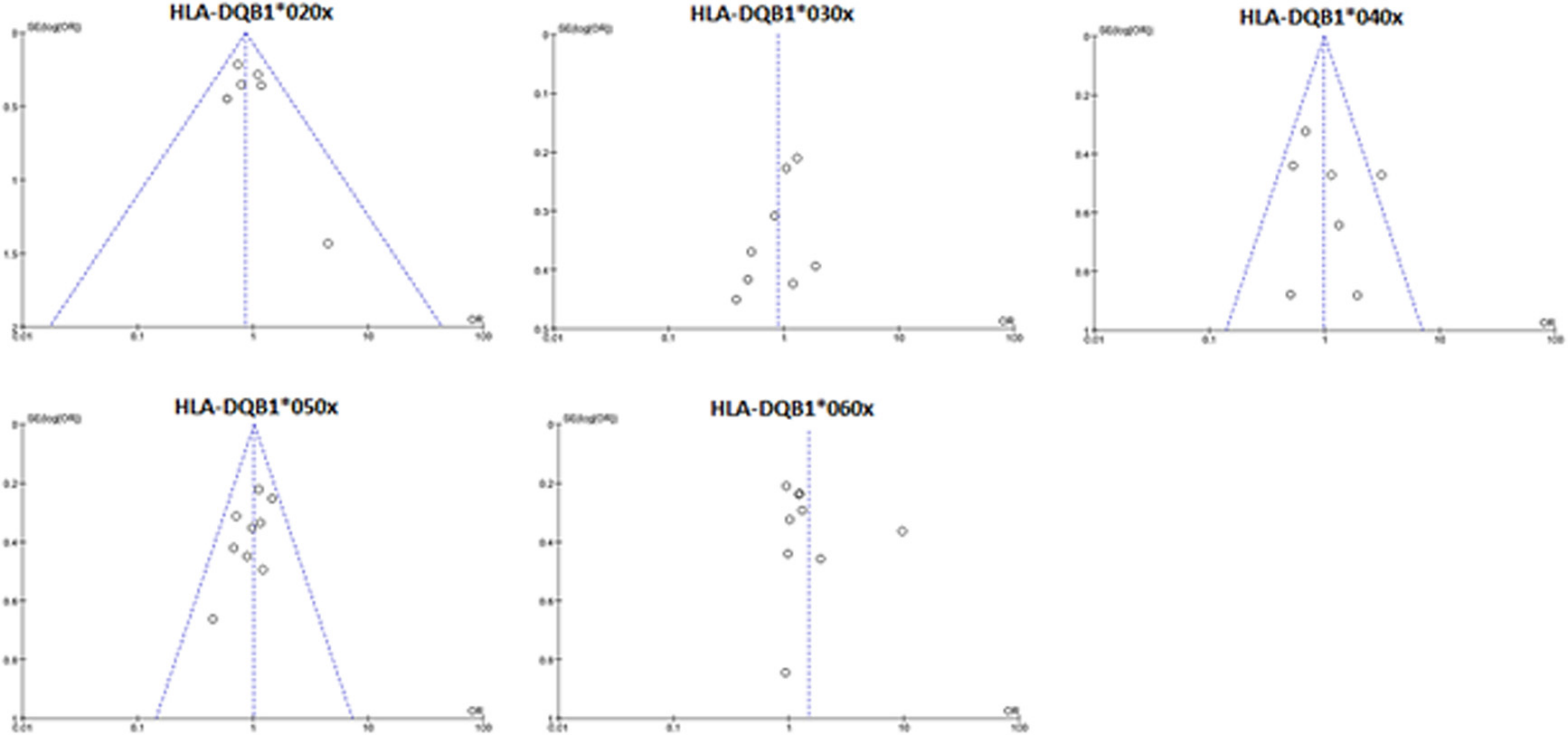
Assessment of publication bias for the HLA-DQB1*020x, *030x, *040x, *050x, and *060x polymorphisms.

Visual inspection of Funnel plots revealed no significant publication bias for the HLA-DQB1 allele studies (Fig 7).

## Discussion

In this study, we investigated the associations between the HLA-DQB1 allele polymorphisms and the risk for GBS. The meta-analysis demonstrated no significant association between any of the HLA-DQB1 alleles and the risk for GBS in a mixed population of Asian and Caucasian patients. There were two associations that approached significance: HLA-DQB1*030x in Asian patients (*P* = 0.07; OR: 0.76, 95% CI: 0.57–1.03) and HLA-DQB1*060x in all patients (*P =* 0.08; OR: 1.48, 95% CI: 0.96–2.29). The heterogeneity observed between studies for the HLA-DQB1*030x polymorphism reflects the mixture of Asian and Caucasian populations, given that this polymorphism is a potential risk factor for Asians but not Caucasians. In contrast, HLA-DQB1*060x only approached significance when all of the studies were utilized, suggesting that its potential association with GBS risk spans ethnic lines. Additional studies are required to determine whether these associations will become significant.

Given that the prevalence of GBS subtypes varies regionally [2], it would be plausible to speculate that this variation is attributable to differences in host immune background and/or local *C*. *jejuni* strains. Our analysis tends to suggest that HLA-DQB1*030x might be one such allele that could contribute to regional variation in GBS. Further studies are warranted to understand how HLA-DQB1*030 and *C*. *jejuni* might interact in Asian populations to affect the risk for GBS.

While we did not identify a significant association between HLA-DQB1 alleles and the risk for GBS, other host factors may still be contributing to GBS risk. The leading hypothesis is that GBS-susceptible hosts produce antibodies targeting bacterial ganglioside-like lipooligosaccharides, which cross-react with gangliosides, leading to axonal degeneration [2, 29, 30]. Host ethnicity may qualitatively affect the way that *C*. *jejuni* strains interact with the immune system to cause different subtypes of GBS [31]. Immunomodulatory host factors may also partially determine the clinical heterogeneity of GBS [32]. Wu *et al*. have conducted a large meta-analysis to assess the contribution of polymorphisms in tumor necrosis factor (TNF)α, FCγrIII, and CD1 to GBS susceptibility. They identified a significant association between the TNFα-308 G/A polymorphism and the risk for GBS, particularly in Asian populations [3]. Thus, polymorphisms in effector molecules likely contribute to GBS susceptibility.

There are also strong ethnic associations between HLA-DR alleles and GBS risk. In Mexican patients, the HLA-DR3 polymorphism has been associated with an increased risk for GBS [33]. In addition, the HLA-DRB1*0701 polymorphism was identified as a novel genetic risk factor for the development of GBS with preceding infection [14]. In Japan, a significantly higher frequency of the HLA-DRBl*0803 polymorphism was found in *C*. *jejuni*-positive GBS patients, compared to controls [26]. Finally, in Dutch GBS patients who needed mechanical ventilation, the frequency of the HLA-DRB1*01 polymorphism was significantly greater than that of controls and patients with less severe disease [24].

There are several limitations to our study. We only investigated the link between HLA-DQB1 alleles and the risk for GBS. Other HLA alleles were not investigated but may contribute to GBS risk and should be evaluated. We were also limited by the relatively small sample size. There is evidence that gender-related factors may influence the interaction between HLA-DR2 polymorphisms and patients with GBS and chronic inflammatory demyelinating polyradiculoneuropathy (CDIP). More female CIDP patients have been reported to be homozygous for HLA-DR2 than male CIDP patients or controls. We were unable to examine the effects of gender in our analysis. Large rigorously conducted studies are needed to elucidate whether there is an association between HLA-DQB1 polymorphisms and the risk for GBS.

In conclusion, our meta-analysis indicated no significant associations between HLA-DQB1 alleles and the risk for GBS in mixed Asian and Caucasian populations. Additional rigorous studies that enroll a large number of patients investigating multiple loci are required to continue investigations into the association between HLA-DQB1 polymorphisms and the risk for GBS.

## Supporting Information

S1 Checklist Meta-analysis on Genetic Association Studies Checklist | Plos ONE. (DOCX)
